# Relationship of High-Density Lipoprotein-Associated Arylesterase Activity to Systolic Heart Failure in Patients with and without Type 2 Diabetes

**DOI:** 10.1038/s41598-019-42518-x

**Published:** 2019-04-12

**Authors:** Chang Li, Jia Wei Chen, Feng Hua Ding, Ying Shen, Zhu Hui Liu, Fang Wang, Rui Yan Zhang, Wei Feng Shen, Lin Lu, Xiao Qun Wang

**Affiliations:** 1 Department of Cardiology, Rui Jin Hospital, Shanghai Jiao-Tong University School of Medicine, Shanghai, P.R. China; 2 Institute of Cardiovascular Diseases, Shanghai Jiao-Tong University School of Medicine, Shanghai, P.R. China

## Abstract

High-density lipoprotein (HDL) confers protection against cardiovascular disease partly attributable to its robust anti-oxidant activities, which is largely impaired in diabetic conditions. In this study, we analyzed the anti-oxidant activity of HDL, as represented by the arylesterase activity of paraoxonase 1 (PON1) in HDL particles, in 216 consecutive HF patients with (n = 79) or without (n = 137) type 2 diabetes, and age- and gender-matched 112 diabetic and 189 non-diabetic non-HF controls. We found arylesterase activity was significantly decreased in patients with than without HF, and was further decreased when comorbid with diabetes. After adjusting for conventional risk factors and apolipoprotein A-I levels, arylesterase activity remained correlated positively with left ventricular ejection fraction in diabetic (r = 0.325, P = 0.020) but not non-diabetic patients (r = 0.089, P = 0.415), and negatively with NT-proBNP and NYHA functional class in both subgroups. In regression analyses, a higher risk of HF was observed in diabetic than non-diabetic patients when having low arylesterase activities. In conclusion, our data demonstrate that impaired serum arylesterase activity in patients with HF is further reduced when comorbid with diabetes. The relationship of impaired arylesterase activity to HF is especially enhanced in diabetic patients.

## Introduction

Patients with diabetes mellitus are at excessive risk of morbidity and mortality of congestive heart failure (HF) than the general population^1–3^. Besides increased coronary dysfunction and atherosclerosis in diabetic conditions, increasing evidence suggests that mechanisms independent of the vasculature, including enhanced oxidative stress, mitochondrial dysfunction, and altered substrate metabolism within the myocardium^4^, play a predominant role in the development of diabetic cardiomyopathy. High-density lipoprotein (HDL) is a well-established cardiovascular protective factor characterized by its reverse cholesterol transport activity as well as robust anti-oxidant and anti-inflammatory properties^5^. While levels of HDL cholesterol (HDL-C), a crude measure of the heterogeneous population of HDL particles, are inversely associated with the risk of cardiovascular events^6,7^, therapies aimed at raising HDL-C concentration failed to improve cardiovascular outcomes in randomized, controlled trails^8,9^. This paradox is partly explained by the findings that HDL quality rather than quantity would be a more relevant measure in association with the risk of cardiovascular events^10,11^, especially under pathophysiological conditions such as diabetes^12,13^. Arylesterase activity, predominantly conferred by HDL-associated paranoxase-1 (PON1), is a typical functional measure reflective of HDL-associated anti-oxidant activities. As one of the most abundant anti-oxidants in the body, PON1 functions to protect low-density lipoprotein (LDL) and HDL from oxidation^14^. Animal studies revealed that PON1 directly reduced oxidative stress in macrophages and the aortic wall^15^. Overexpression of PON clusters was shown to alleviate cardiac hypertrophy in response to angiotensin II^16^. In patients with systolic heart failure, diminished arylesterase activity of HDL was associated with higher risk of incident long-term adverse cardiac event independent of established clinical risk factors^17^. In diabetic conditions, the functional activity of HDL is largely impaired, which may further aggravate the imbalance between pro-oxidant and anti-oxidant substrates within the failing myocardium. Previously, we showed that decreased paraoxonase activity was associated with the presence and severity of coronary artery disease in patients with type 2 diabetes mellitus (T2DM)^18^. In the present study, we analyzed the relationship of seurm arylesterase activity to systolic heart failure in T2DM patients and non-diabetic subjects.

## Results

### Baseline clinical characteristics and biochemical measurements

Baseline characteristics were shown in Table 1. In both diabetic and non-diabetic subjects, patients with HF had lower systolic blood pressure, poorer renal function, lower levels of apolipoprotein A-I, higher incidence of previous myocardial infarction, and more frequently taking diuretics but less frequently statins than non-HF subjects. Compared with diabetic patients without HF, those with HF tended to have higher hsCRP levels. There was no difference in the proportion of patients with CAD and atherosclerosis severity as categorized by the number of diseased vessels among each group. No significant differences between diabetic and non-diabetic patients with HF were observed for left atrial diameter, left ventricular end-diastolic or end-systolic volumes, left ventricular ejection faction (LVEF), N-terminal pro-B-type natriuretic peptide (NT-proBNP) levels, and medication of ACEI/ARB, β-adrenergic receptor blockers, diuretics, statins, and antiplatelet agents.

**Table 1:** Baseline characteristics.

	Non-diabetic patients			Diabetic patients		
	No HF (n = 189)	HF (n = 137)	*P*-value	No HF (n = 112)	HF (n = 79)	*P*-value
Male, n (%)	151 (79.9)	110 (80.3)	0.929	95 (84.8)	69 (87.3)	0.622
Age, years	64.36 ± 11.03	64.31 ± 10.52	0.969	62.45 ± 9.66	62.66 ± 9.77	0.890
Body mass index, kg/m^2^	24.41 ± 3.14	23.83 ± 3.46	0.099	25.47 ± 2.72†	24.61 ± 3.00	0.072
Smoking, n (%)	49 (25.9)	33 (24.1)	0.706	39 (34.8)	23 (29.1)	0.407
Previous myocardial infarction, n (%)	16 (8.5)	24 (17.5)	0.014	7 (6.3)	18 (22.8)	0.001
Hypertension, n (%)	129 (68.3)	69 (50.4)	0.001	81 (72.3)	46 (58.2)	0.042
SBP, mmHg	135.19 ± 19.73	123.46 ± 20.13	<0.001	135.75 ± 20.06	126.13 ± 19.94	0.001
DBP, mmHg	74.88 ± 11.51	74.53 ± 13.20	0.793	77.54 ± 11.76	74.29 ± 12.22	0.069
Fasting glucose, mmol/L	4.87 ± 0.63	4.92 ± 0.71	0.769	6.64 ± 1.91†	6.85 ± 2.04‡	0.283
HbA1c, %	5.70 (5.40–6.00)	5.90 (5.60–6.10)	0.001	7.00 (6.60–7.90)†	7.30 (6.70–8.00) ‡	0.297
HbA1c, mmol/mol	38.80 (35.52–42.08)	40.98 (37.71–43.17)	0.001	53.01(48.63–62.84)	56.28(49.73–63.93)	0.297
Triglyceride, mmol/L	1.26 (0.95–1.76)	1.18 (0.89–1.61)	0.143	1.50 (1.02–2.09)*	1.33 (0.87–1.71)	0.053
Total cholesterol, mmol/L	3.94 ± 1.03	4.07 ± 1.08	0.326	3.88 ± 1.09	3.90 ± 1.17	0.891
HDL cholesterol, mmol/L	1.08 ± 0.27	1.05 ± 0.27	0.297	1.01 ± 0.25*	0.98 ± 0.25	0.482
LDL cholesterol, mmol/L	2.35 ± 0.83	2.45 ± 0.85	0.287	2.25 ± 0.83	2.38 ± 0.91	0.283
Apolipoprotein A-I, g/L	1.28 ± 0.20	1.22 ± 0.21	0.007	1.25 ± 0.20	1.19 ± 0.21	0.049
Apolipoprotein B, g/L	0.79 ± 0.23	0.82 ± 0.23	0.296	0.79 ± 0.24	0.81 ± 0.25	0.521
Lipoprotein (a), g/L	0.15 (0.08–0.31)	0.16 (0.08–0.31)	0.396	0.14 (0.07–0.28)	0.14 (0.07–0.30)	0.874
Uric acid, μmol/L	363.87 ± 98.73	400.30 ± 122.40	0.003	325.44 ± 88.12†	403.30 ± 123.35	<0.001
Blood urea nitrogen, mmol/L	5.50 (4.60–6.53)	6.60 (5.30–8.70)	<0.001	5.50 (4.70–6.68)†	6.20 (5.30–8.53)	0.002
Serum creatinine, μmol/L	84.00 (76.00–97.00)	89.00 (77.00–101.00)	0.084	81.5 (72.00–94.00)*	91.50 (71.75–105.25)	0.034
eGFR, mL/min/1.73 m^2^	78.12 ± 16.15	74.34 ± 17.46	0.046	82.65 ± 17.21*	77.33 ± 20.46	0.039
hsCRP, mg/L	1.14 (0.42–2.55)	1.36 (0.58–7.35)	0.057	0.89 (0.42–2.53)	2.19 (0.53–6.76)	0.008
CAD, n (%)	93 (49.2)	63 (46.0)	0.576	63 (56.3)	42 (53.2)	0.768
1-vessel	46 (24.3)	25 (18.2)	0.222	23 (20.5)	17 (21.5)	0.859
2-vessel	25 (13.2)	17 (12.4)	0.868	22 (19.6)	8 (10.1)	0.105
3-vessel	22 (11.6)	21 (15.3)	0.407	18 (16.1)	17 (21.5)	0.349
multi-vessel disease	47 (24.9)	38 (27.7)	0.610	40 (35.7)	25 (31.6)	0.642
NYHA functional class II/III/IV, n (%)		70/59/8			35/35/9	
NT-proBNP, pg/mL	193.20 (110.95–267.55)	2163.00 (887.80–3531.75)	<0.001	152.05 (84.13–227.83)	1341 (701.03–3009.00)	<0.001
Left atrial diameter, mm	39.0 (36.0–42.0)	47.0 (42.0–50.0)	<0.001	40.0 (38.0–43.0)*	46.0 (43.0–49.0)	<0.001
LVEDD, mm	49.0 (47.0–53.0)	65.0 (59.5–70.0)	<0.001	50.0 (47.0–53.7)	64.00 (61.0–69.0)	<0.001
LVESD, mm	32.00 (29.0–36.0)	54.0 (48.0–59.0)	<0.001	32.0 (29.0–35.0)	53.0 (49.0–58.0)	<0.001
LVEDV, mL	117.0 (102.3–138.0)	214.0 (177.0–257.0)	<0.001	118.0 (104.3–141.5)	209.5 (184.3–263.8)	<0.001
LVESV, mL	41.0 (33.0–54.8)	139.0 (103.5–174.0)	<0.001	41.0 (33.3–52.0)	139.5 (116.0–167.5)	<0.001
LVEF, %	63.0 (60.0–67.0)	35.0 (30.0–38.0)	<0.001	65.0 (60.0–69.0)	34.0 (30.0–38.0)	<0.001
Aspirin, n (%)	134 (70.9)	69 (50.4)	<0.001	72 (64.3)	40 (50.6)	0.059
P2Y12 receptor antagonist, n (%)	96 (50.8)	48 (35.0)	0.005	63 (56.3)	38 (48.1)	0.359
ACE inhibitors or ARBs, n (%)	100 (52.9)	94 (68.6)	0.004	69 (61.6)	58 (73.4)	0.089
Beta-blockers, n (%)	135 (71.4)	103 (75.2)	0.451	75 (67.0)	66 (83.5)	0.010
Nitrates, n (%)	59 (31.2)	39 (28.5)	0.593	35 (31.3)	32 (40.5)	0.187
Statins, n (%)	133 (70.4)	73 (53.3)	0.002	88 (78.6)	50 (63.3)	0.020
Diuretics, n (%)	40 (21.2)	86 (62.8)	<0.001	14 (12.5)	51 (64.6)	<0.001
Oral hypoglycemic drugs, n (%)	—	—		58 (51.8)	33 (41.8)	0.172
Insulin, n (%)	—	—		16 (14.3)	5 (6.3)	0.083

Values are given as mean ± standard deviation, median (interquartile range) or number (percentage).

HF, heart failure; HDL, high-density lipoprotein; SBP, systolic blood pressure; DBP, diastolic blood pressure, LDL, low-density lipoprotein; HbA1c, glycated hemoglobin A1c; NT-proBNP, N-terminal pro-B-type natriuretic peptide; LVEDD, left ventricular end-diastolic diameter; LVESD, left ventricular end-systolic diameter; LVEDV, left ventricular end-diastolic volume; LVESV, left ventricular end-systolic volume; LVEF, left ventricular ejection fraction; eGFR, estimated glomerular filtration rate; hsCRP, high-sentivity C-reactive protein; ACE, angiotensin-converting enzyme, ARB, angiotensin II receptor blocker.

^*^P < 0.05, ^†^P < 0.01, diabetic patients without HF vs. non-diabetic subjects without HF.

^‡^P < 0.01, diabetic patients with HF vs. non-diabetic subjects with HF.

### Serum arylesterase activity in diabetic and non-diabetic HF patients

**Figure 1:**
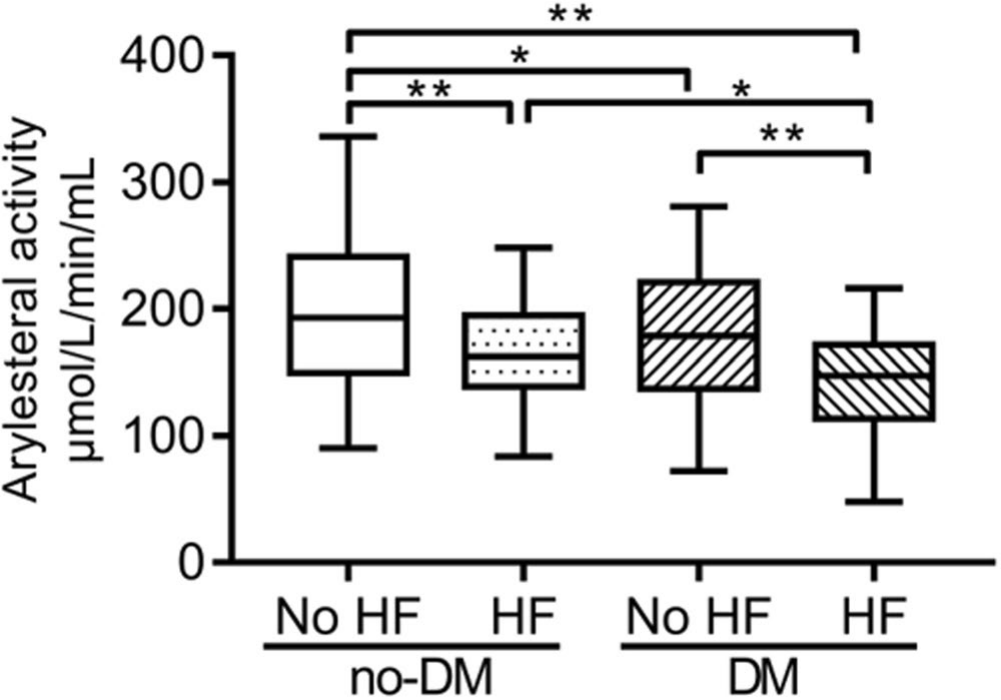
Comparison of serum arylesterase activity among diabetic and non-diabetic patients with or without HF. Diabetic patients tend to have lower arylesterase activity than the non-diabetic controls both in the population with (140.15 ± 45.88 vs. 166.97 ± 49.19 μmol/L/min/mL, P = 0.002) and without HF (179.44 ± 61.40 vs. 199.78 ± 70.51 μmol/L/min/mL, P = 0.005). Serum arylesterase activity is significantly decreased in both diabetic and non-diabetic patients when comorbid with HF. *P < 0.05; **P < 0.01.

Diabetic patients tended to have lower arylesterase activity than the non-diabetic controls both in the population with (140.15 ± 45.88 vs. 166.97 ± 49.19 μmol/L/min/mL, *P* = 0.002) and without HF (179.44 ± 61.40 vs. 199.78 ± 70.51 μmol/L/min/mL, *P* = 0.005). Serum arylesterase activity were significantly decreased in both diabetic and non-diabetic patients when comorbid with HF (Fig. 1). By using two-way ANOVA, arylesterase activity was influenced both by HF (*P* < 0.001) and diabetes (*P* < 0.001), but there is no significant interaction term between heart failure and diabetes (*P* = 0.559, Suppl. Table 1). Among patients with HF, arylesterase activity was not influenced by aetiology of HF (ischemic vs. dilated cardiomyopathy, respectively; *P* = 0.881) but the presence of diabetes (*P* < 0.001, Suppl. Table 2).

**Figure 2:**
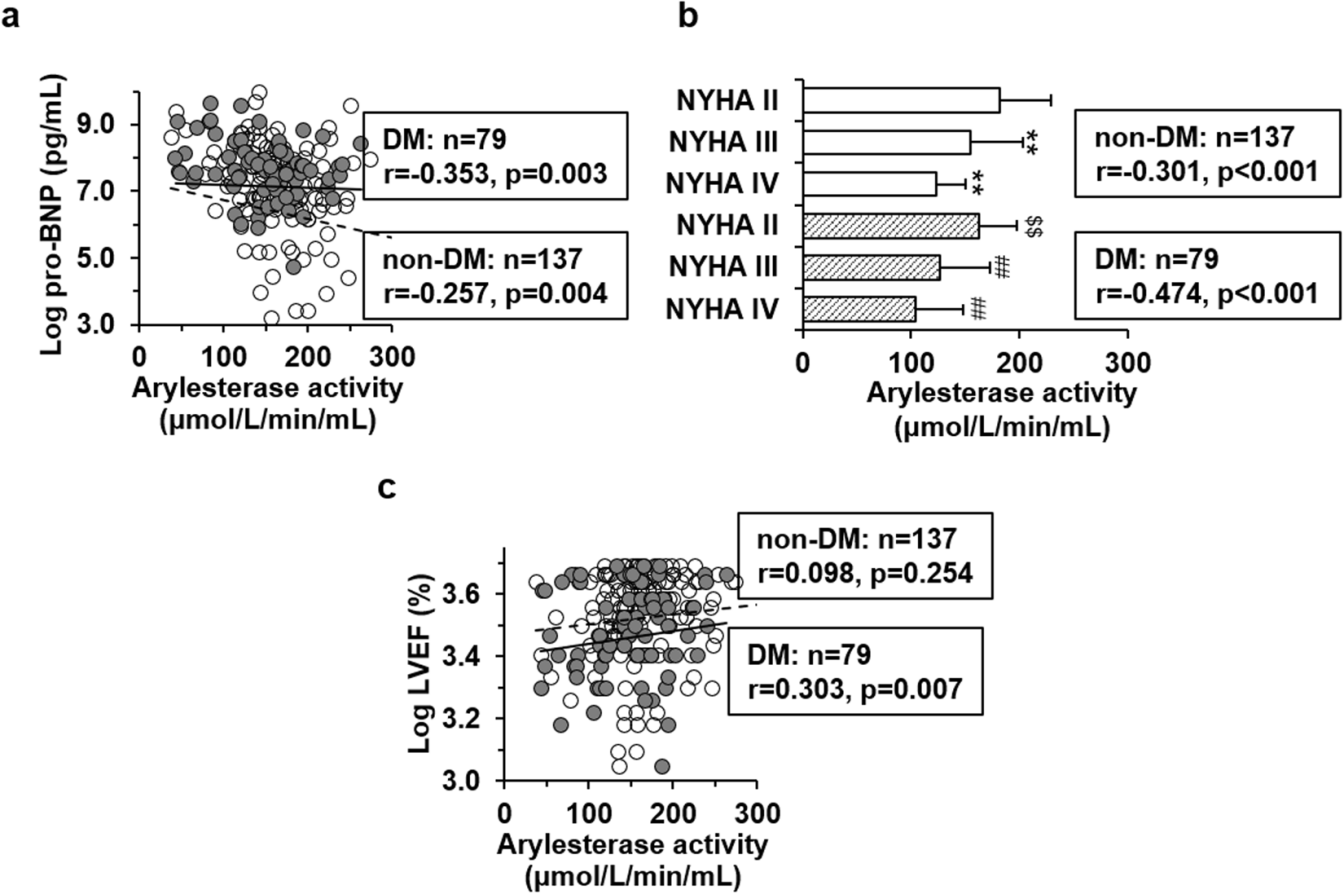
Correlation of serum arylesterase activity with pro-BNP, NYHA functional classes and left ventricular ejection fraction (LVEF) in patients with heart failure. Pro-BNP (**a**) and LVEF (**c**) were logarithmically transformed before plotting. Open dots and dashed line, non-diabetic subjects (n = 137); closed dots and solid line, patients with type 2 diabetes (n = 79). (**b**) Shown is arylesterase activity in non-diabetic (open box) and diabetic (box with diagonal lines) patients grouped by different NYHA functional classes. ^**^p < 0.01 vs. non-diabetic patients with NYHA II functional class; ^##^p < 0.01 vs. diabetic patients with NYHA II functional class; $$p < 0.01 vs. non-diabetic patients with the same NYHA functional class.

In non-diabetic patients with HF, arylesterase activity was associated inversely with ages (r = −0.371, *P* < 0.001), and positively with levels of HDL cholesterol (r = 0.270, *P* = 0.002) and apoA-I (r = 0.257, *P* = 0.004), all of which were attenuated in diabetic subjects (Table 2). Serum arylesterase activity was inversely correlated with log-transformed NT-proBNP levels (non-diabetes: r = −0.257, *P* = 0.004; diabetes: r = −0.353, *P* = 0.003; Fig. 2a) and NYHA functional class (non-diabetes: r = −0.301, *P* < 0.001; diabetes: r = −0.474, *P* < 0.001; Fig. 2b) both in non-diabetic and diabetic subgroups, whereas a positive correlation between arylesterase activity and log-transformed LVEF was only present in diabetic patients (r = 0.303, *P* = 0.007; Fig. 2c). After adjustment for confounding risk factors and apoA-I levels, these correlations remained significant (Table 2).

**Table 2:** Correlation of clinical and laboratory variables with arylesterase activity in heart failure patients

Variables correlated to serum arylesterase activity		Non-diabetic patients with HF		Diabetic patients with HF	
		Correlation coefficient	*P*-value	Correlation coefficient	*P*-value
Unadjusted	Age	−0.371	<0.001	−0.216	0.056
	Log HbA1c	0.035	0.699	0.068	0.554
	Total cholesterol	0.171	0.055	0.212	0.068
	HDL cholesterol	0.270	0.002	0.111	0.344
	ApoA-I	0.257	0.004	0.255	0.028
	eGFR	0.142	0.100	0.190	0.096
	Log NT-proBNP	−0.257	0.004	−0.353	0.003
	Log LVEF	0.098	0.254	0.303	0.007
	NYHA class	−0.301	<0.001	−0.474	<0.001
Adjusted*	Log NT-proBNP	−0.227	0.035	−0.313	0.025
	Log LVEF	0.089	0.415	0.325	0.020
	NYHA class	−0.451	<0.001	−0.554	<0.001

HF, heart failure; HbA1c, glycated hemoglobin A1c; eGFR, estimated glomerular filtration rate; hsCRP, high-sentivity C-reactive protein; NT-proBNP, N-terminal pro-B-type natriuretic peptide; LVEF, left ventricular ejection fraction.

*After adjustment for ages, sex, log transformed hsCRP, eGFR, apo A-I, history of smoking, hypertension and myocardial infarction.

In addition, ROC analysis showed potential superiority of the serum arylesterase activity in evaluating the presence and severity of CHF (NYHA class III-IV) in diabetic (AUC: 0.697, 95% CI: 0.623~0.770, P < 0.001 and AUC: 0.763, 95% CI: 0.659~0.867, P < 0.001, respectively) over non-diabetic patients (AUC: 0.643, 95% CI: 0.584~0.702, P < 0.001 and AUC: 0.652, 95% CI: 0.561~0.743, P = 0.002, respectively).

### Logistic regression analyses

Next, we analyzed the association between arylesterase activity and HF by different regression models. In the univariate analysis, there was a stepwise increase in the odds ratio for HF with decreasing tertiles of arylesterase activity both in diabetic and non-diabetic subjects (p for trend both <0.001). Compared with the highest tertile, the lowest and intermediate tertiles of arylesterase activity correspond to a higher risk of HF in diabetic (7.167- and 5.584-fold, respectively) than non-diabetic patients (3.482- and 2.615-fold, respectively). After adjusting for conventional risk factors including age, sex, history of smoking, hypertension, and previous myocardial infarction (model 2), or with further adjustment for renal function and apoA-I levels (model 3) and medications (model 4), these associations persisted significant in both subgroups. Consistently, the odds ratio for HF in lower tertiles of arylesterase activity tended to be associated with higher risk of HF in diabetic than non-diabetic patients (Table 3).

**Table 3:** Logistic regression analysis of association between arylesterase activity and heart failure.

Arylesterase activity	Non-diabetic patients				Diabetic patients			
	Tertile 1	Tertile 2	Tertile 3	p for trend	Tertile 1	Tertile 2	Tertile 3	p for trend
Model 1^a^	3.482 (1.964–6.174)	2.615 (1.509–4.533)	1	<0.001	7.167 (2.872–17.884)	5.584 (2.183–14.288)	1	<0.001
Model 2^b^	4.063 (2.188–7.547)	2.960 (1.648–5.315)	1	<0.001	7.053 (2.709–18.361)	4.876 (1.835–12.960)	1	<0.001
Model 3^c^	3.727 (1.919–7.240)	2.939 (1.558–5.546)	1	<0.001	5.365 (1.967–14.631)	4.236 (1.566–11.461)	1	0.004
Model 4^d^	4.455 (2.178–9.115)	3.637 (1.827–7.242)	1	<0.001	4.554 (1.613–12.860)	3.846 (1.373–10.770)	1	0.013

^a^Unadjusted.

^b^Adjusted for age, sex, history of smoking, hypertension, and previous myocardial infarction.

^c^Model 2 and additional adjustment for eGFR and apoA-I.

^d^Model 3 and additional adjustment for intake of beta-adrenergic blockers, RAAS inhibitors, and statin.

## Discussion

The major findings of the present study are that impaired serum arylesterase activity in patients with HF is further reduced when comorbid with diabetes. There are no significant interaction effects between heart failure and diabetes on arylesterase activity. The relationship of impaired arylesterase activity to HF is enhanced in diabetic than non-diabetic patients.

Impaired vascular perfusion and low-grade chronic inflammation are pivotal mechanisms contributing to the progression of heart failure. In diabetic patients, the ischemic and inflammatory disorders are usually aggravated due to the presence of metabolic disturbance, diffuse vascular injury, and imbalance between pro-oxidants and anti-oxidants in the myocardium^19^. As a well-established pluripotent atheroprotective factor, the biological function of HDL was shown to be inversely correlated to the risk of cardiovascular disease. However, given the specificity of diabetic cardiomyopathy and markedly impaired functions of HDL in diabetes, it is noteworthy that HDL-associated proteins are subjected to substantial oxidation and glycation under diabetic conditions, which leads to protein dysfunction and the discrepancy between quantity and quality of apoA-I and its associated enzymes. Hence, the beneficial effect of HDL and its role in the risk assessment of heart failure in diabetic patients needs better characterization. In line with previous reports^20^, we showed that the anti-oxidant activity of HDL, as represented by PON1-associated arylesterase activity, was substantially decreased in patients with HF. Especially, the impaired arylesterase activity in diabetic population was further reduced when comorbid with HF. After adjusting for conventional risk factors including apoA-I, arylesterase activity remained significantly correlated to LVEF in diabetic but not non-diabetic patients. ROC analysis further showed that areas under curve of arylesterase activity in evaluating the presence and severity of HF tended to be larger in diabetic than non-diabetic subjects. Finally, the logistic regression analyses demonstrated that the odds ratio for HF was increased with reduced arylesterase levels both in diabetic and non-diabetic patients. Of importance, lower tertiles of arylesterase activity corresponded to a higher risk of HF in diabetic than non-diabetic patients.

Irrespective of the specific aetiology, heightened oxidative stress in diabetes aggravates perturbations of the milieu within the failing myocardium. Treatments targeting at reducing myocardial oxidative damage have provided prospective potential in attenuating cardiomyopathy in diabetic animal studies^21^. Transfer of human ApoA-I to increase HDL levels has effectively reduced the development of streptozotocin-induced diabetic cardiomyopathy^22^. Consistently, our findings suggest that impaired anti-oxidant activity of HDL is associated with, and may also contribute to the development of HF especially in diabetic settings. Paradoxically, the anti-oxidant capability of HDL, as represented by the PON1-associated arylesterase activity, is markedly impaired in diabetic conditions, which may further exacerbate the imbalance between anti-oxidant and pro-oxidant responses in the myocardium and thereby leading to HF progression.

Interestingly, our two-way ANOVA showed no significant interaction term between HF and diabetes on HDL-associated arylesterase activity, suggesting that different mechanisms may additively contribute to the markedly decreased arylesterase activity in diabetic patients with HF. HF is characterized by hemodynamic abnormalities as well as systemic and local inflammation^20,23^. Previously, Kim *et al*. found that serum amyloid A (SAA), a sensitive maker of systemic inflammation, was markedly elevated in HF patients versus controls^20^. Especially, a significant inverse correlation was detected between PON1 activity and SAA levels^20^. Increased SAA associates with HDL, thereby resulting in impairment of its anti-inflammatory properties and cholesterol efflux capacity^20,24^. Additionally, oxidation of HDL was shown to compromise its cholesterol efflux capacity and the stability/activity of its related enzymes^25,26^. Therefore, oxidative stress and inflammatory disorders are likely to be key factors underlying the compromised HDL functional capacity in the setting of HF.

On the other hand, non-enzymatic glycation of HDL plays an essential role for the altered functionality in diabetic conditions^27,28^. *In vivo* studies showed that non-enzymatic glycation of apoA-I impairs its anti-inflammatory properties and the activity of lecithin: cholesterol acyltransferase (LCAT) in mediating cholesterol esterification^29,30^. Previously, we reported that increased apoA-I glycation is associated with the severity of coronary artery disease and plaque progression^18^. Our recent study revealed that apoA-IV, another apolipoprotein within HDL particles, is also subjected to substantial glycation in diabetic patients as assessed by LC-MS analysis^31^. *In vitro* glycation of HDL appears to result in changes of apolipoprotein conformation and thereby decreased functional activity of paraoxonase. Noteworthy, Mastorikou *et al*. reported that glycation of PON1 is increased in patients with diabetes, and *in vitro* glycation of PON1 dramatically reduces its ability to metabolize membrane hydroperoxides^32^. In line with these reports, we showed that PON1-associated arylesterase activity was significantly reduced in diabetic patients regardless of the presence of HF. However, no association was observed between arylesterase activity and glucose or HbA1C levels in diabetic patients with HF. Thus, it is likely that hyperglycemia contributes, but not the solo determinant factor, to the impaired functional activities of PON1 in diabetic patients. Determining the influence of intensive glycemic control and other key factors on the HDL-associated anti-oxidant activities in diabetic patients is important to optimize strategies to restore HDL physiological functions in diabetic conditions.

We recognize limitations in our findings. First, the study was cross-sectional, thereby only allowing us to detect association. Due to the study design, predictions and causal inferences are impossible. Second, the overall sample size was modest and therefore the ability to definitely evaluate the association between HDL function with HF was limited. Third, pre-clinical diastolic cardiac dysfunction is common in patients with diabetes. Further studies focusing on evaluating of HDL function in diabetic patients with ejection fraction-preserved heart failure patients is warranted for the risk stratification at early stage. Fourth, the association of HDL-associated arylesterase activity and adverse cardiovascular outcomes in diabetic patients with HF awaits further investigation. Finally, PON1 possesses paraoxonase activity in addition to the arylesterase activity as measured in the present study. Unlike arlyesterase activity, no association was observed between paraoxonase activity and adverse HF outcomes^33^. However, the potential involvement of paraoxonase activity of PON1 in the pathogenesis of HF, especially in diabetic conditions, cannot be excluded and awaits further characterization.

## Conclusions

This study provides evidence that serum arylesterase activity is reduced both in diabetic and non-diabetic patients with HF. The relationship of arylesterase activity to the presence and severity of HF is enhanced in diabetic than non-diabetic patients. Therefore, diabetic patients with HF is more likely to develop severe HF when having low serum arylesterase activity due to, at least partly, insufficient anti-oxidant capacity. Strategies aimed at restoring the serum arylesterase activity might be useful to counter the over-activated oxidative stress within the myocardium in diabetic patients.

## Methods

This study was approved by the Institutional Review Board of Rui Jin Hospital, Shanghai Jiaotong University School of Medicine, and was registered (NCT02089360). Informed consent was obtained in written form from all patients and clinical investigation was conducted according to the principle of the Declaration of Helsinki.

### Study population

Subjects met the inclusion criteria as follows, (1) symptomatic HF (New York Heart Association class II to IV) for at least 3 months, (2) left ventricular ejection fraction (LVEF) <40%, as documented by echocardiography, within the past 6 months as performed as part of routine care, and (3) age >18 years were enrolled consecutively from the Department of Cardiology, Rui Jin Hospital, Shanghai Jiao-Tong University School of Medicine between February 2014 and June 2016. At last a total of 216 stable systolic HF patients with (n = 79) or without (n = 137) T2DM were enrolled. Another gender- and age-matched 189 non-diabetic and 112 patients with T2DM without HF underwent angiography not in the setting of acute coronary syndrome in Rui Jin hospital during the same period were recruited as respective controls. T2DM was defined according to the American Diabetes Association criteria^34^ as: a fasting plasma glucose levels ≥7.0 mmol/L, or 2-h postprandial plasma glucose readings ≥11.1 mmol/L by multiple determinations, or taking oral hypoglycemic drugs or parenteral insulin. Patients with acute coronary syndrome, primary valvular heart disease, pulmonary heart disease, a history of viral myocarditis, type 1 diabetes, chronic viral or bacterial infections, tumors or immune disorders were excluded. Detailed information of all the patients was obtained on demographics, clinical manifestation, medications, and LVEF determined by echocardiography.

### Biochemical measurements

Peripheral venous blood samples of the patients were collected after an overnight fast after admission to the hospital. Serum glucose, blood urea nitrogen, creatinine, uric acid, triglyceride, total cholesterol, low-density lipoprotein cholesterol, high-density lipoprotein cholesterol, lipoprotein (a), apolipoprotein (apo) A-I, and apo B were measured using standard laboratory techniques on a Hitachi 912 Analyzer (Roche Diagnostics, Mannheim, Germany). The estimated glomerular filtration rate (eGFR) was computed using the Chronic Kidney Disease Epidemiology Collaboration equation^35^. Blood HbA1c concentration was measured using ion-exchange high performance liquid chromatography with Bio-rad Variant Hemoglobin Testing System (Bio-Rad Laboratories, USA). Serum N-terminal pro-B-type natriuretic peptide (NT-proBNP) was determined using a commercially available electrochemiluminescence immunoassay kit (Roche Diagnostics). Serum levels of high-sentivity C-reactive protein (hsCRP) were determined by ELISA (Biocheck Laboratories, Toledo, OH, USA).

### Measurement of serum PON1 arylesterase activity

PON1 arylesterase activity was analyzed from peripheral venous blood samples collected after an overnight fast after admission to the hospital using a photometric assay with phenyl acetate (cat# 108723, Sigma-Aldrich, St Louis, Mo) as the substrate, as previously described^18^. Briefly, initial hydrolysis rates were determined at 270 nm in 100-fold diluted serum (final) in reaction mixtures composed of 10 mmol/L phenyl acetate, 100 mmol/L Tris hydrochloride, pH = 8, and 2 mmol/L calcium chloride at 37 °C. An extinction coefficient (at 270 nm) of 1310/M/cm was used for calculating units of arylesterase activity. PON1 arylesterase activity was monitored in triplicates and the results are presented as μmol/L/min per mL.

### Statistical analyses

Data are expressed as mean ± standard deviation (SD) or median (inter-quartile range) for continuous variables, and frequencies (percentages) for categorical ones. For continuous variables, the existence of normal distribution was ascertained by the Kolmogorov–Smirnov test. Data that were not normally distributed were logarithmically transformed for HbA1c, NT-proBNP, LVEF, hsCRP before analyses were made. Differences among groups were analyzed by one-way analysis of variance (ANOVA) or the Kruskal-Wallis analysis followed by post hoc test with Fisher’s Least Significant Difference (LSD) correction. The Chi-square test was used to compare nominal scale variables. Pearson’s and Spearman’s correlation tests were used to assess the relation between variables. Receiver-operating characteristic (ROC) analyses were used to determine the power of arylesterase activity for detecting the presence or severity of HF and the areas under the curve were compared using the DeLong method^36^.

Different models were established in the regression analysis to evaluate the association between different tertiles of serum arylesterase activity and HF in non-diabetic and diabetic subjects. In model 1, univariate analysis was performed. In model 2, the association was adjusted for conventional risk factors including age, sex, history of smoking, hypertension, and previous myocardial infarction. In model 3, a further adjustment was made for eGFR and apoA-I levels. In model 4, medication status was further adjusted.

All statistical analyses were performed using the SPSS 23.0 for Windows (SPSS, Inc., Chicago, IL, USA). A *P* value < 0.05 was considered statistically significant for all tests including multiple comparisons after LSD correction.

## Supplementary information

Online Supplements

## Data Availability

The study protocol, datasets and statistical code for the current study are available from the corresponding authors on reasonable request.
