# Supplementary material for: Relationship of High-Density Lipoprotein-Associated Arylesterase Activity to Systolic Heart Failure in Patients with and without Type 2 Diabetes
Source: Sci Rep. 2019 Apr 12;9:5979. doi: 10.1038/s41598-019-42518-x (PMC6461629; doi:10.1038/s41598-019-42518-x)
Supplement: Supplementary file 1 — Online Supplements [file 41598_2019_42518_MOESM1_ESM.pdf]

Relationship of High-Density Lipoprotein-Associated Arylesterase Activity to Systolic Heart Failure in Patients with and without Type 2 Diabetes

Chang Li, MD, PhD[1,2]; Jia Wei Chen, MD[1,2]; Feng Hua Ding, MD, PhD[1]; Ying Shen, MD, PhD[1]; Zhu Hui Liu, PhD[1,2]; Fang Wang, MD[1]; Rui Yan Zhang, MD, PhD[1]; Wei Feng Shen, MD, PhD[1,2]; Lin Lu, MD, PhD[1,2*]; Xiao Qun Wang, MD, PhD[1,2*]

Online Supplements

Supplementary table

Supplementary Table 1. Arylesterase activity in diabetic and non-diabetic patients with or without heart failure

	Non-diabetic patients		Diabetic patients		Diabetes	HF	Diabetes × HF
	No HF (n=189)	HF (n=137)	No HF (n=74)	HF (n=79)	*P*1-value	*P*2-value	*P*3-value
Arylesterase activity	199.78 ± 70.51	166.97 ± 49.19	179.44 ± 61.40	140.15 ± 45.88	<0.001	<0.001	0.559

Values are given as mean ± standard deviation. P1, P2, and P3 values stand for the contributions of diabetes, HF, and diabetes × HF interaction to the differences of biological analytes among the four groups by two-way analysis of variance, respectively.

Supplementary Table 2. Arylesterase activity in diabetic and non-diabetic patients with heart failure according to different aetiology

	Ischemic cardiomyopathy (ICM)		Dilated cardiomyopathy (DCM)		Diabetes	Aetiology	Diabetes × aetiology
	Non-diabetes (n=63)	Diabetes (n=42)	Non-diabetes (n=74)	Diabetes (n=37)	*P*1-value	*P*2-value	*P*3-value
Arylesterase activity	167.56 ± 45.67	140.60 ± 44.57	166.47 ± 52.30	139.65 ± 47.94	<0.001	0.881	0.992

Values are given as mean ± standard deviation. *P*1, *P*2, and *P*3 values stand for the contributions of diabetes, aetiology, and diabetes × aetiology interaction to the differences of biological analytes among the four groups by two-way analysis of variance, respectively.
